# Calorimetric determination of the magnetic phase diagram of underdoped ortho II YBa_2_Cu_3_O_6.54_ single crystals

**DOI:** 10.1038/ncomms8927

**Published:** 2015-08-21

**Authors:** C. Marcenat, A. Demuer, K. Beauvois, B. Michon, A. Grockowiak, R. Liang, W. Hardy, D. A. Bonn, T. Klein

**Affiliations:** 1 Université Grenoble Alpes, INAC-SPSMS, F-38000 Grenoble, France; 2 CEA, INAC-SPSMS, F-38000 Grenoble, France; 3 Université Grenoble Alpes, LNCMI, F-38042 Grenoble, France; 4 CNRS, LNCMI, F-38042 Grenoble, France; 5 Université Grenoble Alpes, Institut NEEL, F-38042 Grenoble, France; 6 CNRS, Institut NEEL, F-38042 Grenoble, France; 7 National High Magnetic Field Laboratory, Florida State University, Tallahassee, Florida 32310, USA; 8 Department of Physics and Astronomy, University of British Columbia, Vancouver, British Columbia, Canada V6T 1Z1; 9 Canadian Institute for Advanced Research, Toronto, Ontario, Canada M5G 1Z8

## Abstract

The recent discovery of a charge order in underdoped YBa_2_Cu_3_O_*y*_ raised the question of the interplay between superconductivity and this competing phase. Understanding the normal state of high-temperature superconductors is now an essential step towards the description of the pairing mechanism in those materials and determining the upper critical field is therefore of fundamental importance. We present here a calorimetric determination of the field–temperature phase diagram in underdoped YBa_2_Cu_3_O_*y*_ single crystals. We show that the specific heat saturates in high magnetic fields. This saturation is consistent with a normal state without any significant superconducting contribution and a total Sommerfeld coefficient *γ*_N_∼6.5±1.5 mJ mol^−1^ K^−2^ putting strong constraints on the theoretical models for the Fermi surface reconstruction.

## Abstract (web summary)

The presence of a charge order state in underdoped YBCO raises the question of the interplay between this phase and the superconducting one. Here, the authors characterize this material's phase diagram through specific heat and magnetic measurements, providing strong constrains to theoretical models.

## Graphical abstract

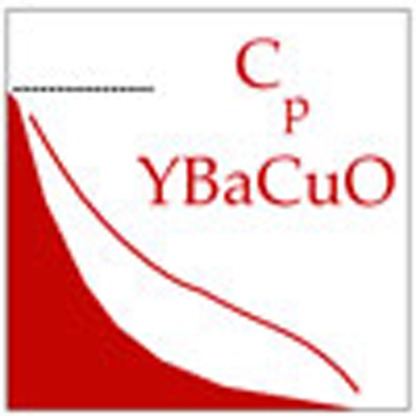

The discovery of a charge order (CO) phase in underdoped YBa_2_Cu_3_O_*y*_ (YBCO)^1^,^2^,^3^,^4^,^5^,^6^,^7^ associated to a major Fermi surface reconstruction^8^,^9^,^10^,^11^,^12^ revived the debate on the pairing mechanism in high-temperature superconductors, still one of the most challenging issue in modern solid-state physics. The normal state has recently been the focus of intensive works^8^,^9^,^10^,^11^,^12^,^13^,^14^,^15^,^16^,^17^ but the detailed nature of this state remains controversial. A sharp feature in the thermal conductivity^13^ has been ascribed to the onset of vortex scattering at the superconducting to normal state transition and the observation that the Wiedemann–Franz law is well obeyed above this characteristic field^17^ clearly underlined that the system becomes fully metallic without any trace of superconductivity. However, a small diamagnetic component has recently been observed well above 25 T (ref. 14) and specific heat measurements displayed a square root of *H* dependence of the Sommerfeld coefficient all the way up to 45 T (ref. 15), both suggesting that superconductivity may persist up to much larger magnetic fields in agreement with some theoretical predictions (see for instance ref. 18 and references therein).

We focused on samples for which the oxygen atoms in the CuO chains are fully ordered in the so-called ortho-II structure^19^ (*y*∼6.54) corresponding to a hole doping *P*∼0.11 and *T*_c_∼62 K. A first consequence of the competition between superconductivity and CO is a drastic reduction of the upper critical field *H*_c2_(0) for this doping content^13^, offering the unique opportunity among cuprates to study the phase diagram down to the lowest temperatures. To address the crucial issue of the existence of a superconducting state well above the resistive transition, we have performed highly sensitive specific heat measurements from 7 to 70 K and up to 26 T. Calorimetry is indeed a very powerful tool for the detection of thermodynamic lines and we show that a clear specific heat anomaly is visible in both temperature (at low field) and magnetic (at low temperatures) sweeps. The specific heat saturates in high magnetic fields with a total Sommerfeld coefficient *γ*_N_∼6.5±1.5 mJ mol^−1^ K^−2^ putting strong constraints on the theoretical models for the Fermi surface reconstruction. This saturation is also consistent with a normal state without any significant superconducting contribution, in contradiction to previous experimental results^15^.

## Results

### Specific heat measurement

**Figure 1:**
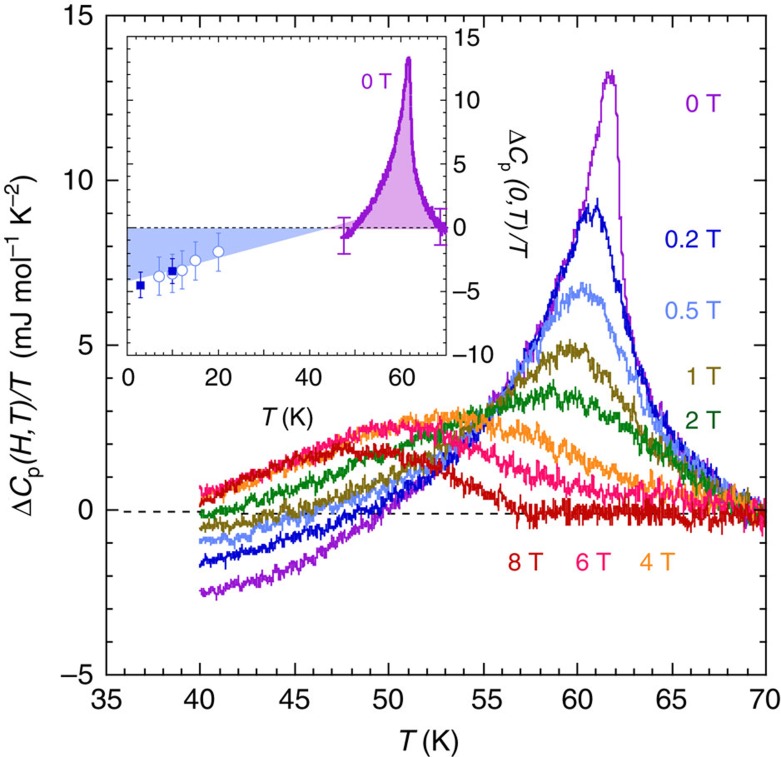
Specific heat of YBa_2_Cu_3_O_6.54_. Temperature dependence of the specific heat for the indicated magnetic fields 
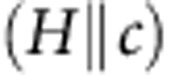
 with Δ*C*_p_(*H*, *T*)=*C*_p_(*H*, *T*)−*C*_p_(*H*_max_, *T*) and *H*_max_=14 T. The temperature *T*_Cp_ corresponding to the maximum of the specific heat anomaly has been reported in Fig. 2 (solid (red) symbols). The shape of the anomaly and its evolution in magnetic field are characteristics of the superconducting transition in the presence of large thermal fluctuations. Inset: Δ*C*_p_(0,*T*)/*T* as a function of *T* from the 0-T temperature sweep (purple line, same as main panel, *H*_max_=14 T) and the magnetic field sweeps displayed in Fig. 3 (blue symbols, *H*_max_=26 T). The solid squares have been deduced from relaxation data and the open circles from the modulation technique data (see Fig. 3). As shown, the two sets of data are consistent with the entropy balance law as the blue and purple areas are identical. The error bars represent the systematic uncertainties, were estimated along the different calibrations steps, and are dominated by the field dependence of the addenda contribution.

In all samples, a clear specific heat anomaly is visible for *H*=0 at the superconducting transition temperature *T*_c_∼62 K. Note that its amplitude corresponds to ∼1% of the total specific heat, which is dominated by the phonon contribution. To overcome the problem to estimate this lattice contribution, we have subtracted from all curves the data obtained at 14 T (see Fig. 1), a high enough field to strongly suppress most of the anomaly, hence being a good approximation of the normal state specific heat in this temperature range. Even if a smooth background can still be present below 50–55 K, measurements above 20 T (not shown for clarity) confirmed that the specific heat is field independent above 14 T (and *T*>40 K) within our error bars and increased noise in resistive magnets. A two times smaller anomaly has been previously reported by Loram *et al.*^20^ in polycrystalline samples of similar composition. This zero-field *C*_p_ anomaly presents the characteristic shape of a superconducting transition in presence of strong thermal fluctuations with significant departures from the classical mean-field theory^21^ and is consistent with thermal expansion data^22^. A strong positive curvature is visible on both sides of the transition and a large fluctuation contribution to the specific heat is measured up to ∼10 K above *T*_c_. The overall shape of the anomaly is intermediate between the highly asymmetric jump measured in optimally doped YBCO, and the symmetric cusps with no underlying mean-field contribution observed in very anisotropic materials such as Bi-2212 and Hg-1223 (ref. 21). Note that the amplitude of the anomaly is drastically reduced by magnetic field, dropping by a factor 2 for fields as small as 0.5 T. As expected in presence of strong thermal fluctuations, *C*_p_/*T* follows a logarithmic decay in field at *T*=*T*_c_(0), in very good agreement with previous measurements in optimally doped YBCO and highly anisotropic systems^21^.

**Figure 2:**
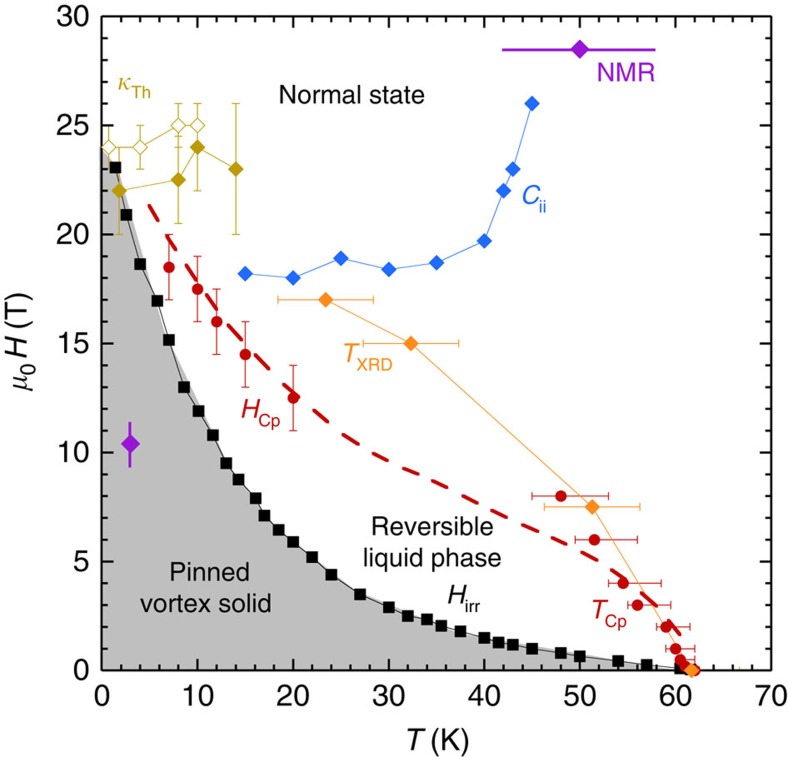
*H*–*T* phase diagram of YBa_2_Cu_3_O_6.54_. The solid (red) circles correspond to the position of the specific heat anomaly (maximum or onset of saturation, see Figs 1, 3) and the solid (black) squares the position of the irreversibility line (*H*_irr_) deduced from the onset of harmonics in the magnetic susceptibility (see Fig. 4). Characteristic lines deduced from other probes have also been reported for comparison; *c*_ii_, onset of the sound velocity anomaly from ref. 4; *κ*_Th_, onset of the thermal conductivity drop from ref. 13 (longitudinal *κ*_xx_, closed symbols) and ref. 17 (transverse *κ*_xy_, open symbols); NMR, splitting of the nuclear magnetic resonance peaks from ref. 1 and ref. 33; *T*_XRD_, cusp in the amplitude of the CO diffraction peaks from ref. 3 (the temperatures have been rescaled to take into account the slight change in *T*_c_ in this *y*∼6.67 sample). The error bars are as reported in the corresponding references. The dashed (red) line corresponds to the locus of the upper critical field deduced from *H*_irr_(*T*) in the melting theory^28^,^29^ with 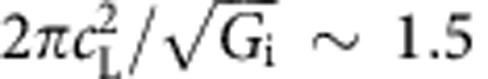, that is, corresponding to a very reasonable Lindemann coefficient *c*_L_∼0.22 for a Ginzburg parameter *G*_i_∼5.10^−2^ (ref. 27).

The *C*_p_ anomaly is then shifted towards lower temperatures for increasing magnetic fields. We believe that the maximum indicates the transformation of the vortex-liquid state into the normal state as the well-defined phase transition marked by a specific heat singularity transforms into a gradual crossover in the presence of strong fluctuations. This situation is similar of the maximum in *C*_p_ observed when crossing the widom line emanating from the critical point in supercritical fluids^23^. The location of the maximum then divides the supercritical region into gas-like and liquid-like domains, reminiscent of subcritical well-defined thermodynamic phases. The position of this maximum (*T*_Cp_) in our data has been reported in Fig. 2. To give an indication of the width of the crossover, we have also reported an error bar corresponding to the difference between the inflexion point and the maximum itself. However, for either of those two criteria, a classical Werthamer–Helfand–Hohenberg extrapolation would lead to a *T*=0 characteristic field significantly higher than the 24±2 T value deduced from thermal conductivity and electrical and thermal Hall conductivity measurements^13^, indicating a renormalization (reduction) at low temperature.

**Figure 3:**
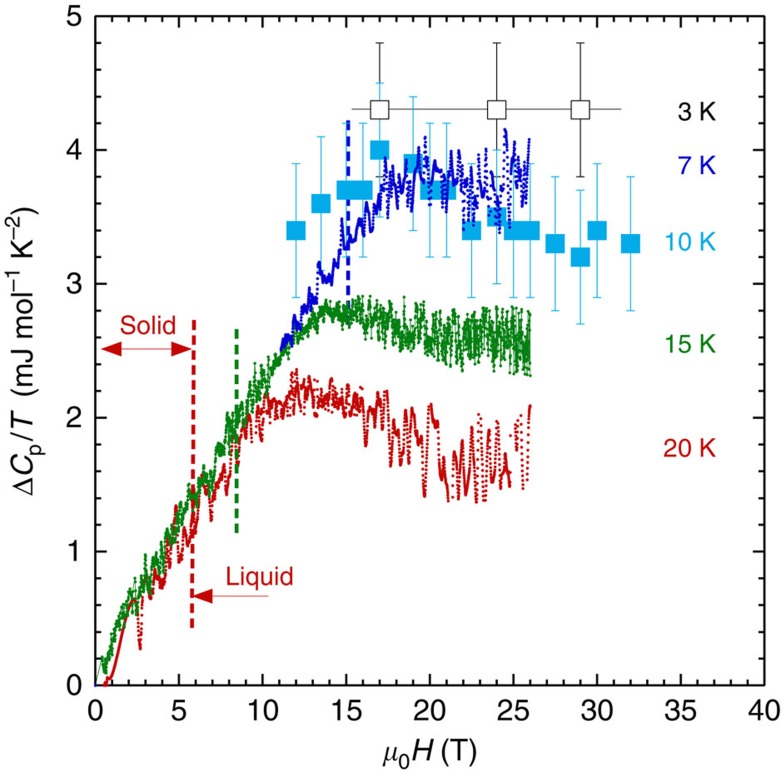
Magnetic field dependence of the specific heat in YBa_2_Cu_3_O_6.54_. Δ*C*_p_(*H*, *T*)/*T*=*C*_p_(*H*, *T*)/*T*−*C*_p_(0, *T*)/*T* initially increases almost linearly with field due to the increase of the number of vortices in the mixed state and flattens off for large magnetic fields. Solid lines correspond to the modulation technique results (at the indicated temperatures) and the solid (blue) squares at 10 K have been obtained using a relaxation technique. The (purple) squares at 3 K correspond to the difference 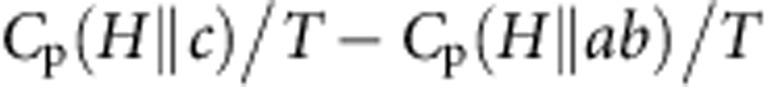 between data obtained for both field orientations (see text for details). The vertical dotted lines mark the position of the irreversibility line deduced from magnetic measurements (see Fig. 4) corresponding to the solid/liquid transition line. As shown, the specific heat anomaly clearly lies in the vortex liquid and is therefore not related to the melting of the vortex solid. The *H*_Cp_ values corresponding to the maximum of *C*_p_ (or onset of saturation al low temperature) have been reported in Fig. 2 (solid (red) circles). The error bars are systematic uncertainties obtained from the calibration measurements performed on ultrapure copper.

To extend the magnetic phase diagram to lower temperatures, we have performed field sweeps at fixed temperatures down to 7 K (see Fig. 3). *C*_p_/*T* initially increases almost linearly with field due to the increase of the number of vortices in the mixed state and flattens off in large magnetic field. As for the *T*-sweeps, a (broad) maximum is visible above 15 K and the observed field dependences are again generic of the superconducting to normal state crossover in the presence of strong thermal fluctuations. Both the initial linear dependence and the final saturation differ from the lower temperature data^15^, which displayed a 
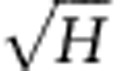
 dependence of the Sommerfeld coefficient up to 45 T. Such a field dependence is expected to be observed in d-wave superconductors due to a Doppler shift of the quasiparticule excitation spectrum around the vortex cores^24^ but only well below a crossover temperature for which the thermal energy is smaller than the Doppler energy. This difference can then be due to the fact that our measurements have been performed in a *T*_c_/9≤*T*≤*T*_c_/3 temperature range, whereas the data presented in ref. 15 were taken below 2 K (∼*T*_c_/30). We checked carefully that the saturation of the specific heat at high magnetic field is independent of the history of the sample (zero-field cooled or field cooled), as well as of the sweeping rate and size of the temperature oscillations.

### Field–temperature *H*–*T* phase diagram

**Figure 4:**
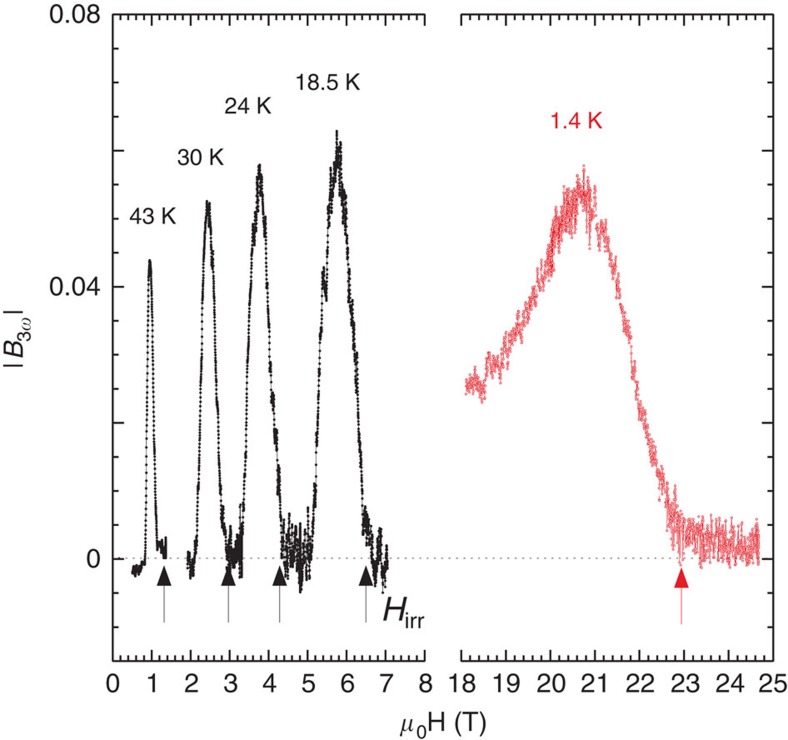
Third harmonic component of the local magnetic field. Main panel: magnetic field dependence of the amplitude of the third harmonic component of the local magnetic field 
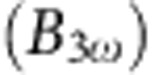
 detected by a miniature Hall sensor and normalized to full screening. The sharp onset of 
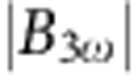
 marks the onset of the nonlinear response of the vortex matter at the irreversibility line *H*=*H*_irr_ corresponding to the melting of the vortex solid (marked by the arrows). The corresponding line has been reported in Fig. 2 (solid (black) squares). Measurements have been performed on a platelet-like crystal (black symbols, see also Figs 1, 3 for specific heat data on this sample) as well as on a slab of a larger crystal of same composition (red symbols, see Methods for further details).

As for the *T*-sweeps, the magnetic fields corresponding to the *C*_p_/*T* anomaly (*H*_Cp_, onset of saturation or maximum for *T*>15 K) have been reported on Fig. 2. Unfortunately, the phonon contribution to the specific heat rapidly rises with temperature and the relative change in field drops below 0.5% above 20 K hindering any determination of the *C*_p_/*T* anomaly for intermediate temperatures. To verify that the maximum of *C*_p_ is not related to the melting of the vortex matter we have performed magnetic measurements on the same single crystals. The detection of the third harmonic component of the local magnetic field 
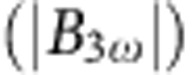
 is one of the most reliable criterion to determine the irreversibility line as 
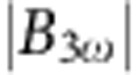
 abruptly rises at the transition from a reversible (liquid) to an irreversible (solid) state^25^,^26^, as a consequence of the nonlinear response of vortices in the pinned solid state (see Fig. 4). As shown in Fig. 2 (see also Fig. 3), the *H*_Cp_ line lies well above the magnetic irreversible field, that is, being in the vortex-liquid region.

### Sommerfeld coefficient

As shown in Fig. 3, the modulation calorimetry data yield *C*_p_(*H*_max_)/*T*−*C*_p_(0)/*T*≈4±1 mJ mol^−1^ K^−2^ at low temperature. We also confirmed this value using the relaxation technique on a larger sample (solid squares in Fig. 3). Finally, to eliminate possible spurious field-dependent contributions such as a Schottky-like anomaly^15^, we have also performed relaxations for 
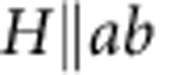
 on the same sample. Owing to the strong anisotropy of the superconducting phase^27^ the data obtained for this field direction can be used as a baseline including all potential extra (isotropic) contributions to the specific heat. We hence confirmed that 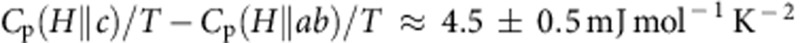 at 3 K (for *H*>17 T). Finally, note that the Δ*C*_p_/*T* values measured at low temperature are consistent with the entropy balance law that can be estimated from the two sets of *C*_p_/*T* data (see inset of Fig. 1), showing that the entropy does not depend on field at 70 K. Finally, we measured a residual Sommerfeld *γ*_0_ contribution in zero field on the order of 2.5±0.5 mJ mol^−1^ K^−2^ in good agreement with the value previously obtained by Riggs *et al.*^15^. The total normal state value *γ*_N_=*γ*_0_+Δ*C*_p_(*H*)/*T* is hence on the order of 6.5±1.5 mJ mol^−1^ K^−2^.

## Discussion

The characteristic lines deduced from different experimental criteria have been reported in Fig. 2. As shown, our data are in agreement above 50 K with the *T*_XRD_ line (orange diamonds) below which the CO is suppressed by the onset of superconductivity, as detected by X-ray diffraction^3^. As *T* tends to zero, both our *H*_Cp_ line and the one deduced from thermal conductivity measurements (brown diamonds)^13^ converge towards the same characteristic field ∼24±2 T. The fact that the specific heat is independent of field and that the Wiedemann–Franz law is obeyed above this field is a compelling evidence that the system becomes fully metallic with no significant superconducting phase. At low temperature, our *H*_Cp_ line also lies close to the one deduced from sound velocity measurements^4^ (blue diamonds) but with significant differences in their temperature dependencies. In the absence of clear objective criteria it is however difficult to point the exact location of a phase transition in the data presented here, especially in this complex part of the phase diagram. Nevertheless, below 20 K, *H*_Cp_ is very close to the inflexion point in the field dependence of *c*_11_. The authors of ref. 4 rather reported the onset of the vortex lattice softening, which would then better corresponds to the ill-defined field above which the specific heat saturates.

It is also worth mentioning that the maxima detected at high temperature (*T*_Cp_, *T*-sweeps) and at low temperature (*H*_Cp_, *H*-sweeps) can actually be connected by a single line calculated from the irreversibility line in a phenomenological description of the vortex melting using a Lindeman criterion^28^,^29^,^30^ (see dashed line in Fig. 3 and corresponding text in the figure caption). A reasonable scenario would then be that thermal conductivity and specific heat track the two bounds of the same crossover of the vortex liquid into the normal state. The thermal conductivity being very sensitive to the scattering of vortices entering into the sample would detect the upper bound of the transition, whereas the specific heat being sensitive to all thermal excitations would mark the lower bound. However, the weak and smeared features in *C*_p_ hinders any definite conclusion from our data on a scenario for which two well-defined thermodynamic transitions would occur, a field-induced charge ordering detected by sound velocity measurements and an upper critical field ascribed to the anomaly in thermal conductivity.

Finally, let us discuss the absolute value of the field-induced change in *C*_p_/*T* at low temperature. In a two-dimensional reconstruction model involving pockets of effective mass *m*_i_ and multiplicity *n*_i_, the Sommerfeld coefficient is expected to be equal to 1.45 mJ mol^−1^ K^2^ × 
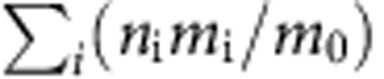
 (per CuO_2_ planes) and introducing^31^ two hole pockets of effective mass *m*_h_∼0.45±0.1*m*_0_ and one electron pocket of effective mass *m*_e_=1.7±0.2*m*_0_ (ref. 16) one expects *γ*_N_=7.6±0.8 mJ mol^−1^ K^−2^ in good agreement with our experimental value. However, this agreement requires to ascribe the origin of the zero field *γ*_0_ to pair-breaking effects inducing a large density of states at zero energy in the nodal direction of the order parameter. A scattering rate Γ/Δ_0_∼0.01−0.1 (Δ_0_ being the maximum amplitude of the superconducting gap) could account for *γ*_0_/*γ*_N_∼1/3 and could also explain the observed breakdown of the scaling of the vortex specific heat with 
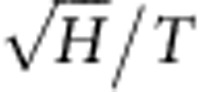
 (see ref. 24 and reference therein). However, such a large scattering rate seems to be inconsistent with the observation of quantum oscillations^8^,^12^ and with the linear temperature dependence of the superfluid density^32^ suggesting that the samples are in a clean limit with a much smaller Γ/Δ_0_ ratio. Finally, note that the residual Sommerfeld *γ*_0_ value is strikingly constant, varying only between ∼2 and ∼2.5 mJ mol^−1^ K^−2^ among the best crystals with optimum, overdoped and ortho II doping^24^ suggesting that this contribution is actually not related to pair-breaking effects, hence implying that the relevant figure that has to be compared with the predictions of the reconstruction models is 4±1 mJ mol^−1^ K^−2^. Complementary experiments are needed to clarify the origin of this residual Sommerfeld coefficient.

## Methods

### Specific heat

*C*_p_ measurements have been performed in magnetic fields up to 26 T using an high-sensitivity modulation technique. Heat was supplied to the sample by a light-emitting diode via an optical fibre and the induced temperature oscillations (*T*_ac_∼500 mK r.m.s. at 1 Hz) were recorded with chromel–constantan thermocouples. Systematic checks were carried out to ensure that the results were independent of the amplitude of the temperature oscillations, of the magnetic history (zero field cooled or field cooled), and of sweeping rates. Calibrations were made *in situ* by measuring a piece of copper (6N) of about the same mass and geometric aspect as those of the samples. These measurements were done on three platelet-like crystals grown at the University of British Columbia (UBC)^19^ with mass *m∼*0.2–0.3 mg. A relaxation technique has been used at low temperature (below 10 K and up to 34 T) for the determination of the absolute value of the heat capacity. The chip resistance of the micro-calorimeter used as both the thermometer and the heater, together with the thermal conductance of its leads, has been carefully calibrated using a capacitance thermometer. Each relaxation provides about 1,000 data points over a temperature interval of about 50% above the base temperature, which has been varied between 2 and 10 K. Data can be recorded during heating and cooling. The merging of the upward and downward relaxation data provides a highly reliable check of the accuracy of this method.

### Magnetic measurements

The oscillating component of the local induction has been recorded by centring the samples on miniature GaAs-based quantum well Hall sensors. The third harmonic component of the local field (
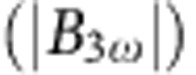
) has been recorded as a function of field for fixed temperatures in presence of a modulation field applied perpendicularly to the Hall probe (*h*_ac_∼1*G*, *ω*∼200 Hz). Measurements have been performed on the platelet-like crystals used for calorimetric measurements (black symbols in Fig. 4) as well as on a slab of a larger crystal of same composition (red symbols in Fig. 4). In this latter case, the static field 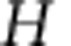 was applied perpendicularly to *h*_ac_ (for example, parallel to the probe), whereas for the platelets both *H* and *h*_ac_ were applied perpendicularly to the probe 
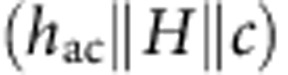
. Both geometries are probing the onset of a sustainable current at the irreversibility line but the former one enables to reduce the noise associated with the very large constant component present in the second geometry. This geometry could hence be used to detect the anomaly up to very large static fields (see for instance Fig. 4 for data up to 26 T at 1.4 K).

## Additional information

**How to cite this article:** Marcenat, C. *et al.* Calorimetric determination of the magnetic phase diagram of underdoped ortho II YBa_2_Cu_3_O_6.54_ single crystals. *Nat. Commun.* 6:7927 doi: 10.1038/ncomms8927 (2015).
